# Structured Ternary Fluids Promote Crystallization and Detection of 5‑Methyl-2-[(2-nitrophenyl)amino]-3-thiophenecarbonitrile (ROY) Metastable Polymorphs

**DOI:** 10.1021/acs.cgd.5c00838

**Published:** 2025-11-04

**Authors:** Yang Shu, Jennifer J. Maunder, Nicci L. Fröhlich, Madeleine Rolle-Binne, Sharon J. Cooper

**Affiliations:** Department of Chemistry, 3057 Durham University, Durham DH1 3LE, U.K.

## Abstract

Polymorph screens are typically resource- and time-intensive, requiring multiple solvent systems. Here, we report that structured ternary fluids (STFs), dynamic analogues of microemulsions, facilitate the simultaneous crystallization of multiple 5-methyl-2-[(2-nitrophenyl)­amino]-3-thiophenecarbonitrile (ROY) polymorphs from a single composition, despite the dominance of the stable Y form as both the thermodynamic and kinetic product. Previously, we demonstrated that STFs enable selective crystallization of all three ambient-pressure polymorphs of glycine. Here, we extend this approach to ROY, using toluene–water–isopropanol STFs to access metastable polymorphs that remain elusive in conventional toluene–isopropanol mixtures. Additionally, at high supersaturations, an amorphous phase can initially precipitate before being consumed by crystalline forms. We attribute these findings to the ability of STFs to maintain elevated local supersaturations and to generate higher nucleation rates and lower crystal growth rates than conventional solvents. A new STF pipetting perturbation strategy, after sedimentation of the first-formed Y prisms, promotes selective autocatalytic secondary nucleation of the metastable polymorphs, facilitating their isolation. This study reveals the potential of STFs for rapid polymorph screening.

## Graphical abstract

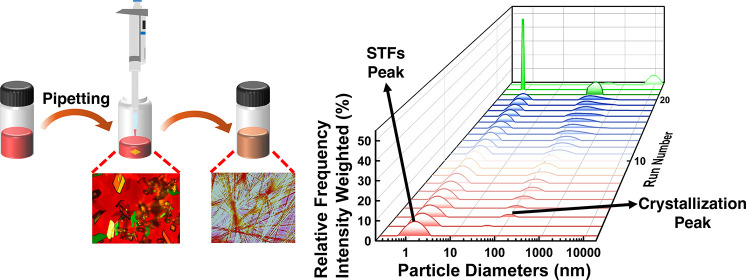

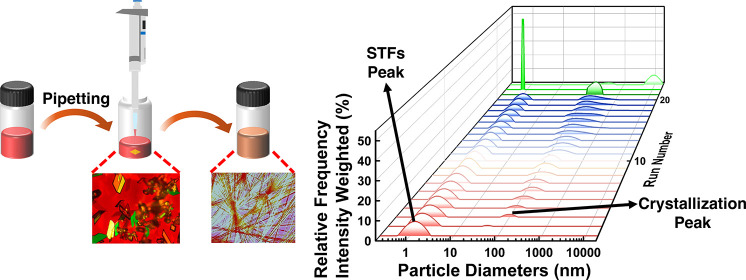

## 1 Introduction

Polymorph screening is essential to try to ensure the optimum product for the over 80% of chemicals, and 90% of pharmaceuticals that are manufactured in the crystalline state. A small polymorph screen typically involves solubility studies in 20 to 30 different solvents and 3–4 different crystallization methods, while a larger screen requires over 300 experiments.^1^ However, even after this effort, the polymorph screen may fail to find the stable polymorph because crystallization control in bulk solution is typically under kinetic control. Consequently, current polymorph screening strategies are often wasteful in resources, time and money and may fail to locate useful low energy polymorphs.

Crystallization in microemulsions can ensure production of the stable polymorph because the nanoconfinement provides a methodology to switch from kinetic to thermodynamic control.^2–5^ However, the approach can be limited by too slow crystal growth, which can restrict crystal sizes to submicrometres, and by contamination from difficult-to-remove surfactants. Recently, we demonstrated that nanoconfinement-induced thermodynamic control of crystallization is also possible in structured ternary fluids (STFs),^6^ with these systems offering significant advantages: the crystallization kinetics are no longer prohibitively slow and the crystals obtained are impurity-free.

STFs consist of two immiscible liquids, typically an oil and water, and an amphisolvent that is miscible with both. The first STF was reported in 1977 but the nanostructuring present was only fully elucidated more recently using small-angle X-ray and neutron scattering,^7–12^ and static and dynamic light scattering.^13^ Corroborating evidence has also been provided by NMR, conductivity, UV–vis spectrometry of probe molecules and molecular dynamics simulations.^14–18^ At low and high oil contents, STFs contain dynamic ∼2–5 nm-sized pockets of oil-in-water (o/w) or water-in-oil (w/o), respectively, while bicontinuous structures with channel widths ∼2–10 nm arise when the oil and aqueous quantities are similar.

The presence of these ∼2–10 nm domains has led to STFs often being described as surfactant-free microemulsions or ultraflexible microemulsions. However, we advocate for the term structured ternary fluids (STFs) because the nanostructuring is inherently more dynamic and flexible than in conventional microemulsions, where surfactant geometry and chemistry impose a preferred curvature and hence a more rigid constraint on droplet size. Furthermore, surfactants exhibit a critical micelle concentration and are strongly anchored to the interfacial region. In contrast, the amphisolvent in STFs exhibits only a modest affinity for the interfacial region, being readily soluble in both the oil and aqueous phases. Notably, upon further addition of the amphisolvent, the nanostructuring dissipates to give a homogeneous, fully mixed solution–this is diametrically opposed to the effect in microemulsions, where lyotropic liquid crystalline order can emerge upon addition of more surfactant.

Our previous study on glycine crystallization in an octanol–water–ethanol STF demonstrated that polymorph control is achievable within a single STF composition by varying the supersaturation because the glycine is largely confined to the aqueous pockets, with restricted glycine diffusion between these pockets.^6^ Accordingly, at low supersaturation, thermodynamic control prevailed with γ-glycine dominating, despite the well-known difficulty of crystallizing this polymorph from aqueous solution. At intermediate supersaturations, α-glycine is the majority polymorph, while at high supersaturations, the unstable β-form could be nucleated, facilitated by the increased local supersaturation heterogeneity in STFs, and then easily isolated because the restricted glycine diffusion between aqueous pockets slowed down the Ostwald ripening dissolution of this form. Furthermore, this restricted diffusion produced unique high nucleation rate/low crystal growth kinetics that significantly prolonged the lifetime of nanoscale nuclei.

In this study, we investigate whether STF nanoconfinement can promote the appearance and persistence of metastable polymorphs of 5-methyl-2-[(2-nitrophenyl)­amino]-3-thiophenecarbonitrile, which is commonly known as ROY because of its Red, Orange and Yellow polymorphs. ROY is widely regarded as a model system for polymorph screening due to it having the largest number of known polymorphs–14 to-date.^19^ The Y prism form is the thermodynamically stable form under ambient conditions with the known metastable forms lying within 6.6 kJ mol^–1^.^20,21^ We selected an STF comprising toluene, water, and 52.5 wt % isopropanol (IPA) because ROY is highly soluble in toluene but virtually insoluble in water. Consequently, at low wt % toluene, ROY is largely confined to toluene-rich nanometre-sized droplets, with only restricted ROY diffusion between these droplets. This system was also particularly suitable for exploring whether STFs promote crystallization of metastable polymorphs because it is inherently biased against such outcomes: the stable Y prism form of ROY is typically the sole product in conventional cooling crystallizations using toluene and the binary mixture of 47.5 wt % toluene: 52.5 wt % IPA. This is because it usually appears first and so is both the kinetic and thermodynamic product. Notably, here we use a new strategy involving STF perturbation through postnucleation mechanical disturbance to maximize the detection of metastable polymorphs.

## 2 Materials and Methods

### 2.1 Materials

The chemicals used were as follows: toluene (≥99.8%, Fisher Scientific), isopropyl alcohol (≥99.5%, Fisher Scientific), 5-methyl-2-[(2-nitrophenyl)­amino]-3-thiophenecarbonitrile (ROY) (Sigma-Aldrich), methyl orange (Fisher Scientific). Ultrahigh purity water (18.2 MΩ cm) was obtained from a Sartorius arium comfort water purifier.

### 2.2 Capillary Viscometry

The viscosity of the fluids was determined using an *Ubbelohde* Dilution Viscometer by measuring the time taken for flow between two points for the fluid samples and a water reference.

### 2.3 Dynamic Light Scattering (DLS)

Solutions were filtered through 0.1 μm filters into 1 cm^3^ quartz cuvettes. DLS measurements were performed using an Anton Paar Litesizer 500 using 90° side scattering. The normalized autocorrelation function, *g* ^(2)^(*τ*), was measured as a function of delay time, *τ*, and automatically fitted using the standard cumulant analysis method^22^ to output mean droplet sizes calculated from the Rayleigh Stokes equation using the inputted viscosity value. For the *in situ* ROY crystallization experiments, the quartz cuvette and filtration equipment were heated alongside the sample to prevent crystallization occurring prior to DLS measurements.

### 2.4 UV–Vis Spectroscopy

Samples were placed in a *Unicam* UV2 UV–vis spectrometer and scanned between 300–600 at 240 nm min^–1^ at 1 nm intervals. A methyl-orange (1.79 mmol dm^–3^) containing sample and reference sample with the same composition but without the methyl orange were prepared. The methyl orange containing sample was then diluted with the reference sample until its absorbance *A* < 2.0 before obtaining the absorbance spectrum.

### 2.5 Solubility of ROY in the STFs and Binary Solution

STFs containing toluene, water and IPA were prepared for the required compositions on a 5 g scale. Excess ROY was then weighed and added to each sample vial, which were then sealed using PTFE tape. Each composition was made twice, with each duplicate being stored either in a fridge at 7 °C or a water bath at 25 °C for two months. After this time, the residual ROY was extracted via Büchner filtration. To ensure there is no remaining filtrate on the residual ROY, the ROY is rinsed with a few ml of ethanol, and then water, followed by drying overnight in an oven at 50 °C. The residual ROY is weighed to determine the mass dissolved.

### 2.6 ROY Crystallizations

Binary systems comprised of 47.5 wt % toluene and 52.5 wt % IPA, and STFs comprised of 52.5 wt % IPA and varying proportions of toluene and water, were made on a 5 g scale. ROY was added to each sample to achieve the required relative supersaturation, *c*/*c*_sat_, at 25 °C, where *c* is the ROY concentration and *c*_sat_ is its saturation concentration (Table S1). Sample vials were sealed with PTFE tape before being heated to 65 °C on a hot plate until all the ROY dissolved. Samples were then placed in an oven at 70 °C for at least an hour to ensure full ROY dissolution. The samples were removed from the oven, placed in a water bath at 25 °C and left to crystallize either undisturbed, or were perturbed by one of two methods: shaking for five seconds or pipetting 0.5 cm^3^ of solution out and back into the upper half of the solution three times in quick succession. Crystals were extracted from solution via Büchner filtration. In the initial crystallization trial, crystals were extracted when a metastable polymorph was observed in the sample vial or after 1 week if no metastable forms were visible. In subsequent crystallizations, crystals were extracted either at timed intervals or when a metastable polymorph was observed.

### 2.7 Optical Microscopy

Optical microscopy data were acquired on an Olympus BX50 microscope equipped with a digital camera. Samples were transferred to a clean glass microscope slide before images were taken. To assess crystal growth rates, standard glass microscope slides were cut in half before spreading silicone grease around the outside to form a border. 2–3 drops of fluid were then transferred to the center of one of the prepared slides before sealing with the other half of the slide, pressing down lightly to remove any air gaps. The sealed sample was then placed on a Linkam heating/cooling block, which was fitted with a central hole to allow light transmission through the sample. The sample was heated to 70 °C to ensure all ROY remained dissolved, before cooling to 25 °C, where the temperature was held. After the first crystal was identified, images were taken once every minute, with the length and width of the crystal(s) in each sequential image then measured. The increase in circumference per unit time was calculated, and from this, the crystal growth rate was determined.

## 3 Results and Discussion

### 3.1 STF Verification

The existence of the toluene-water-IPA STF was previously proposed based on NMR, conductivity and DLS studies of compositions with constant water mole fraction.^23^ Our more detailed DLS studies revealed that for 52.5 wt % IPA compositions (Figure 1a), the decay of the autocorrelation function, *g* ^(2)^(*τ*)-1, yielded correlation times of ∼10–20 μs (Figure 1b,c), consistent with ∼1–2 nm structuring (Table S2), thereby confirming STF formation. However, this nanostructuring was lost at higher IPA contents (Figure 1d,e). Consequently, all crystallization studies were performed using compositions containing 52.5 wt % IPA. The DLS correlation times provide a lower bound for the nanopocket lifetimes; if lifetimes were shorter than this, *g* ^(2)^(*τ*)-1 would not exhibit time-dependent decay consistent with intact diffusing nanopockets. Hence, we anticipate nucleation and crystal growth mechanisms to be impacted by nanoconfinement on at least this 10–20 μs time scale.

**Figure 1:**
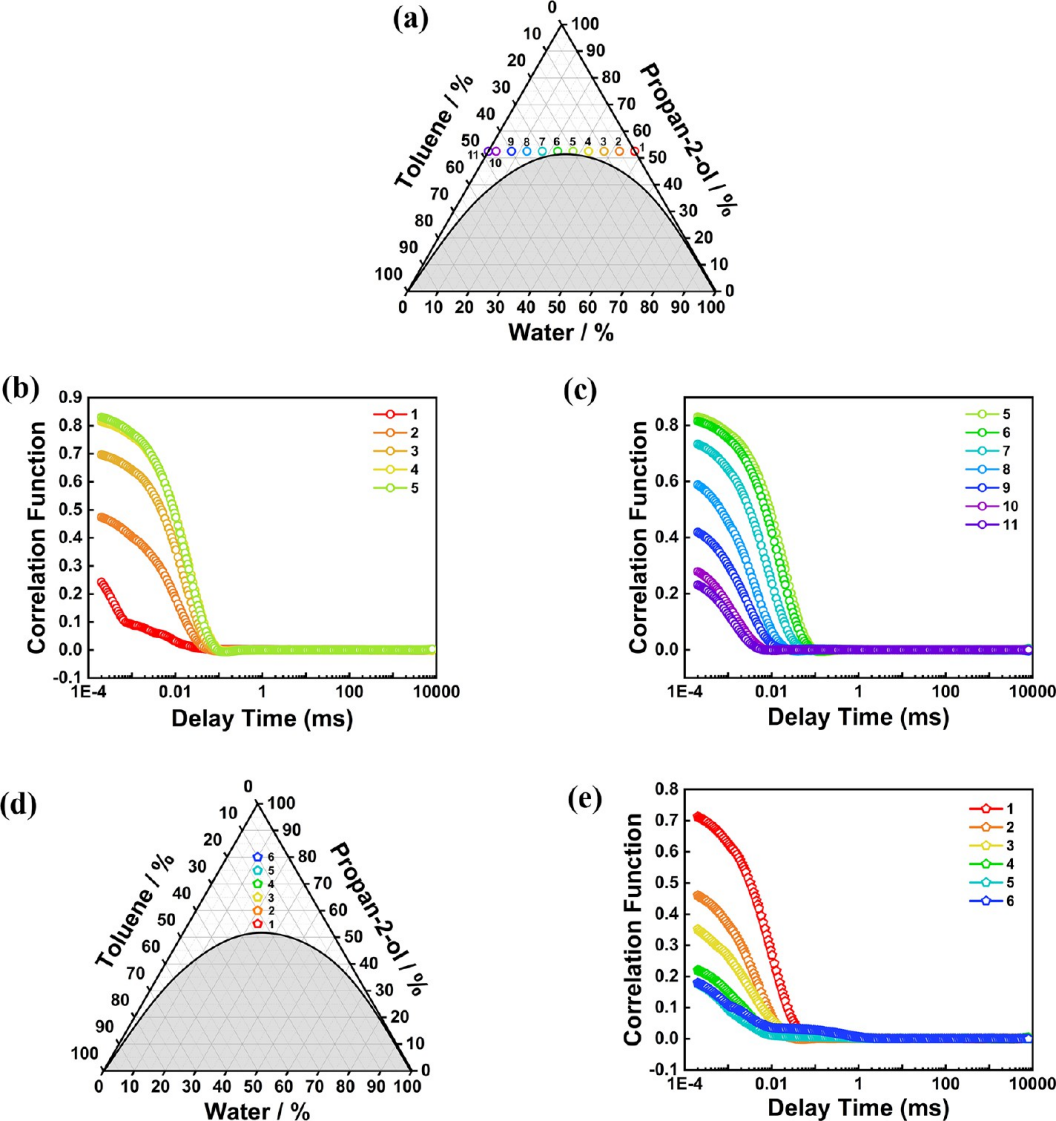
(a, d) Ternary phase diagrams of toluene–water–IPA by percentage mass with numbered symbols showing the compositions probed by DLS. (b, c, e) Time-dependent autocorrelation functions, *g* ^(2)^(*τ*)-1, measured by DLS.

UV–vis spectroscopy studies (Figure 2) using the water-soluble dye methyl orange showed the expected increase in its absorption maximum as the proportion of water increased. The small size of the aqueous pockets in the w/o STFs and the narrow aqueous channels in the bicontinuous STFs prevent complete shielding of methyl orange from the surrounding toluene phase. As a result, the dye experiences a less polar environment and exhibits a lower absorption maximum than when fully solvated in the larger aqueous domains of o/w STFs. The increase in absorption maximum with rising water content is least pronounced in the bicontinuous region due to the more gradual swelling of the aqueous channel width compared to the more rapid enlargement of aqueous pocket size at lower aqueous contents. The faster increase in absorption maximum in the o/w STFs reflects the rapid loss of interfacial area reducing methyl orange contact with toluene. In comparison, the 75 wt % IPA unstructured ternary solutions produced a featureless decline in absorption maximum with increasing toluene wt % (Figure 2 inset). These UV–vis spectroscopy findings further confirm the STF nature of the 52.5 wt % IPA-toluene-water compositions.

**Figure 2:**
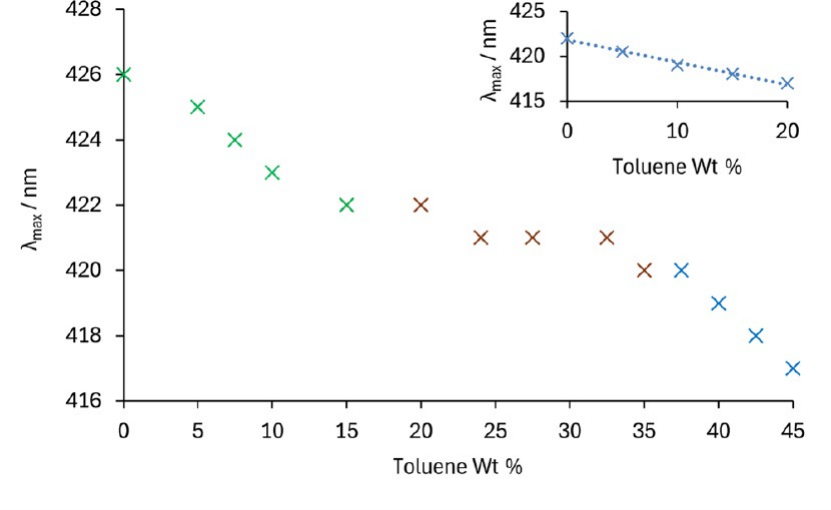
Wavelength of absorption maximum of methyl orange, *λ*_max_, as a function of toluene wt % in STFs containing 52.5 wt % IPA and a binary 47.5 wt % toluene: 52.5 wt % IPA solution. o/w (green), bicontinuous (brown), and w/o (blue) regions are indicated. The inset shows values obtained for 75 wt % IPA solutions, which lack nanostructuring.

### 3.2 Initial Crystallization Studies

Initial crystallization studies were undertaken to establish the supersaturation ranges and STF compositions most likely to promote metastable polymorphs of ROY (Table 1). These established that while the Y prism form was the dominant polymorph, and typically appeared first, metastable YN, ON and R forms did arise, and on rarer occasions occurred in the absence of the Y prism form. The likelihood of metastable crystals appearing increased with relative supersaturations *c*/*c*_sat_ ≥ 3, where *c* is the ROY concentration and *c*_sat_ is its saturation concentration (Table S1). Though less frequent, the four crystal polymorphs, Y, YN, ON and R could also appear in a single crystallization experiment for the 5–10 wt % toluene STFs, particularly at the higher supersaturation of *c*/*c*_sat_ = 5.5 (Figure 3).

**Table 1:**
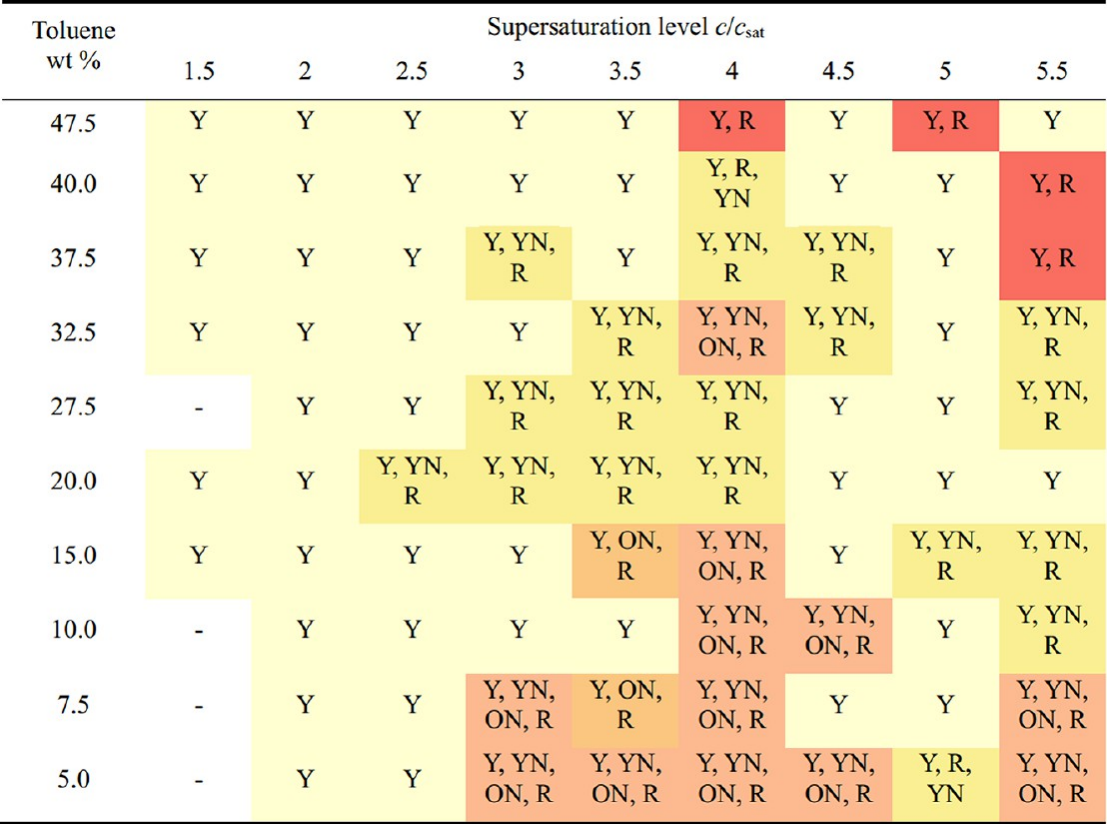
Polymorph Outcome from Crystallizations, with Extraction after the Appearance of a Metastable Polymorph or after 1 Week If No Metastable Polymorphs Appeared

**Figure 3:**
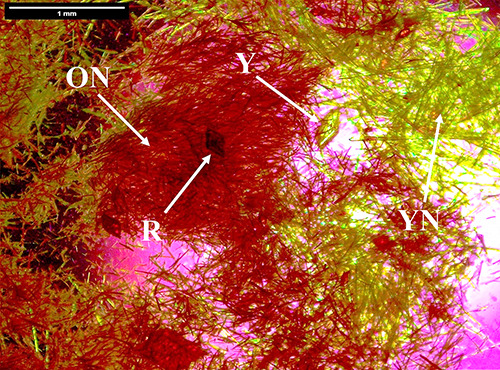
*c*/*c*_sat_ = 5.5 ROY crystallization from a 5 wt % toluene STF showing crystallization of Y prisms, YN, ON, and R polymorphs.

To investigate the probability of metastable polymorph observation further, crystallization experiments with time-varying filtrations were conducted at a lower temperature of 7 °C to access higher supersaturations up to *c*/*c*_sat_ values of 12 (Table 2). The first timed filtrations consistently yielded only the Y form. Metastable forms emerged in the second timed filtrations because sufficient time had elapsed for their nucleation and growth to detectable dimensions. At later filtrations, metastable forms were less prevalent, and sometimes absent (e.g., the 2-h filtration of *c*/*c*_sat_ = 5.5 5 wt % toluene STF), since Ostwald ripening had depleted these forms so that only Y prisms remained. The much higher supersaturation levels of *c*/*c*_sat_ = 11 and 12 did not increase the production of metastable forms, probably because rapid Y prism crystallization depleted the supersaturation to lower levels not conducive to their nucleation.

**Table 2:**
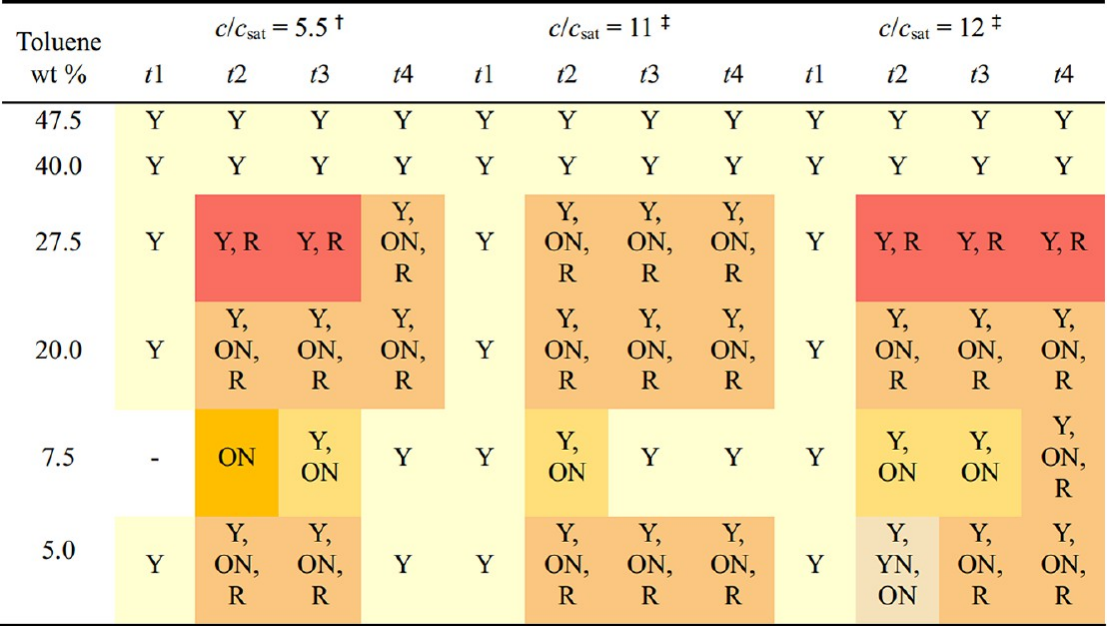
Polymorph Outcome after Timed Extractions^a^,^b^

a †: Timed extractions every 30 min.

b ‡: Timed extractions every 30 min for 5 and 7.5 wt % toluene samples and every 3 min for the remainder.

Notably, in one instance involving the second timed filtration with 5 wt % toluene, the YN metastable form was observed. The YN polymorph has a significantly higher free energy, and so greater solubility, than the R, ON, and Y forms, thereby requiring a much higher supersaturation relative to the Y form for its nucleation. Such a high local supersaturation would be unlikely to persist in unstructured solutions, as elevated ROY concentrations would be rapidly dissipated by unrestricted diffusion. In contrast, restricted ROY diffusion between nanoconfined STF pockets allows locally high concentrations to persist for extended periods. Thus, nanoconfinement can promote crystallization of otherwise unfavored forms by sustaining the higher supersaturation conditions necessary for their nucleation.

### 3.3 Crystal Growth Studies

The slower ROY crystal growth in the 5 wt % toluene STF was tracked through *in situ* crystallization studies using optical microscopy. Figure 4 shows optical microscopy images for crystal growth comparisons in *c*/*c*_sat_ = 3.0 systems, while Figure 5 and Figure S1 show growth comparisons at *c*/*c*_sat_ = 5.5 using optical microscopy images and growth rate data, respectively. The markedly slower crystal growth in o/w STFs is further demonstrated in comparative videos constructed from time-lapse optical microscopy images (Videos S1–S6). The restricted diffusion responsible for the slow crystal growth also significantly hinders Ostwald ripening, increasing the likelihood that metastable polymorphs persist for longer. Given the appearance of metastable polymorphs in the filtrate, we hypothesized that these longer-lived metastable forms would have a greater chance of being observed if the STF structure was perturbed at opportune times to selectively enhance further secondary nucleation of these forms. Secondary nucleation is known to dominate over primary nucleation in stirred crystallizations and this can be used advantageously to induce deracemization.^24,25^

**Figure 4:**
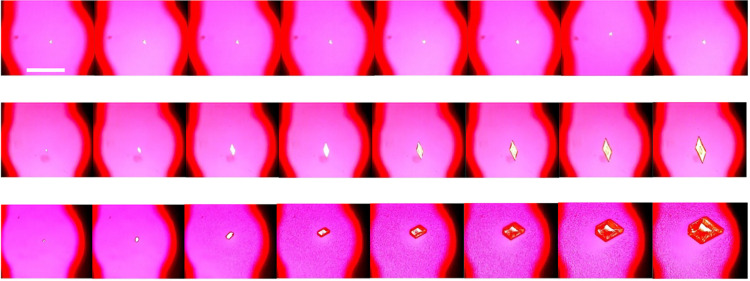
Crystal growth comparisons for *c*/*c*_sat_ = 3.0 supersaturations. Top = 5 wt % toluene STF, middle = 20 wt % toluene STF, bottom = binary toluene:IPA solution. Consecutive images are 20 s apart. The droplets in the binary images are the amorphous phase. Images taken through crossed polars with a red tint plate. Scale bar = 1 mm.

**Figure 5:**
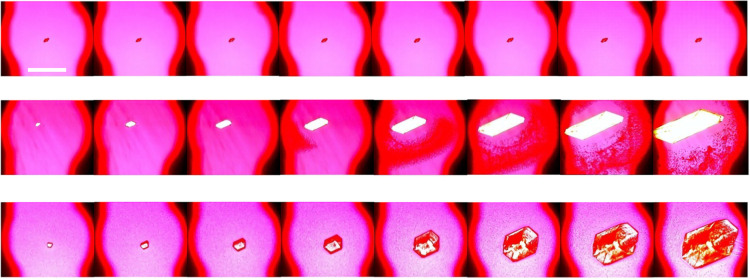
Crystal growth comparisons for *c*/*c*_sat_ = 5.5 supersaturations. 5 wt % toluene STF (top), 20 wt % toluene STF (middle), binary toluene:IPA solution (bottom). Consecutive images are 10 s apart. The droplets in the 20 wt % STF and binary system images are the amorphous phase. Images were taken through crossed polars with a red tint plate. Scale bar = 1 mm.

### 3.4 Perturbation Studies

Comparison experiments were conducted on nanoconfined ROY STFs to assess how frequently metastable polymorphs were observed with and without disturbance of the STF. Two different STF perturbation methods were applied: (i) 5 s of shaking or (ii) pipetting 0.5 cm^3^ of solution from and back into the upper half of the solution three times. In both cases, the perturbation was conducted after sedimentation of the typically first-formed Y prisms. The shaking strategy was expected to induce a second burst of Y prisms via disturbance, fragmentation and autocatalytic secondary nucleation of the sedimented Y prisms. In contrast, the pipetting strategy was anticipated to primarily promote autocatalytic secondary nucleation of any suspended nuclei and therefore targeted the metastable forms. At *c*/*c*_sat_ = 4.0, Y prisms sedimented within ∼10 min to 1 h for STFs containing >10 wt % toluene, whereas for lower toluene contents, sedimentation required several hours or overnight. Following either pipetting or shaking, new metastable crystals typically emerged almost immediately or within 5–30 min. Pipetting following initial Y prism sedimentation significantly increased the proportion of metastable forms compared to the undisturbed controls (mean difference = +0.38, Tukey *post* *hoc* *p* < 0.0001), whereas shaking was ineffective (*p* = 0.8125) (Figure 6). Representative images of YN and ON polymorphs produced by the pipetting are shown in Figure 7.

**Figure 6:**
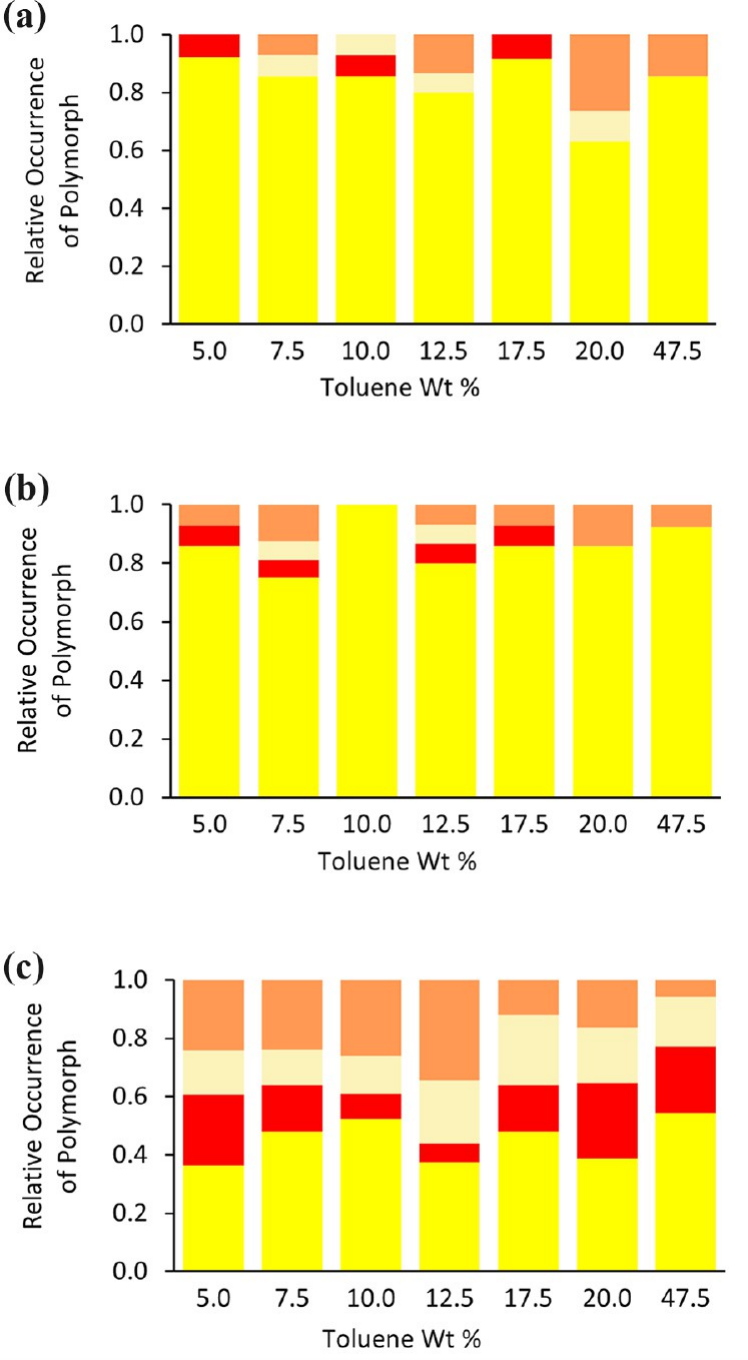
Relative occurrence of ROY polymorphs in ROY *c*/*c*_sat_ = 4.0 STFs and the binary IPA:toluene solution for (a) 12 repeated crystallizations with samples left undisturbed, (b) 12 repeated crystallizations shaken for 5 s after Y prisms' first sedimentation, and (c) 12 repeated crystallizations with pipetting three times after Y prisms' first sedimentation. Y prisms (yellow), YN (pale yellow), ON (orange), and R (red).

**Figure 7:**
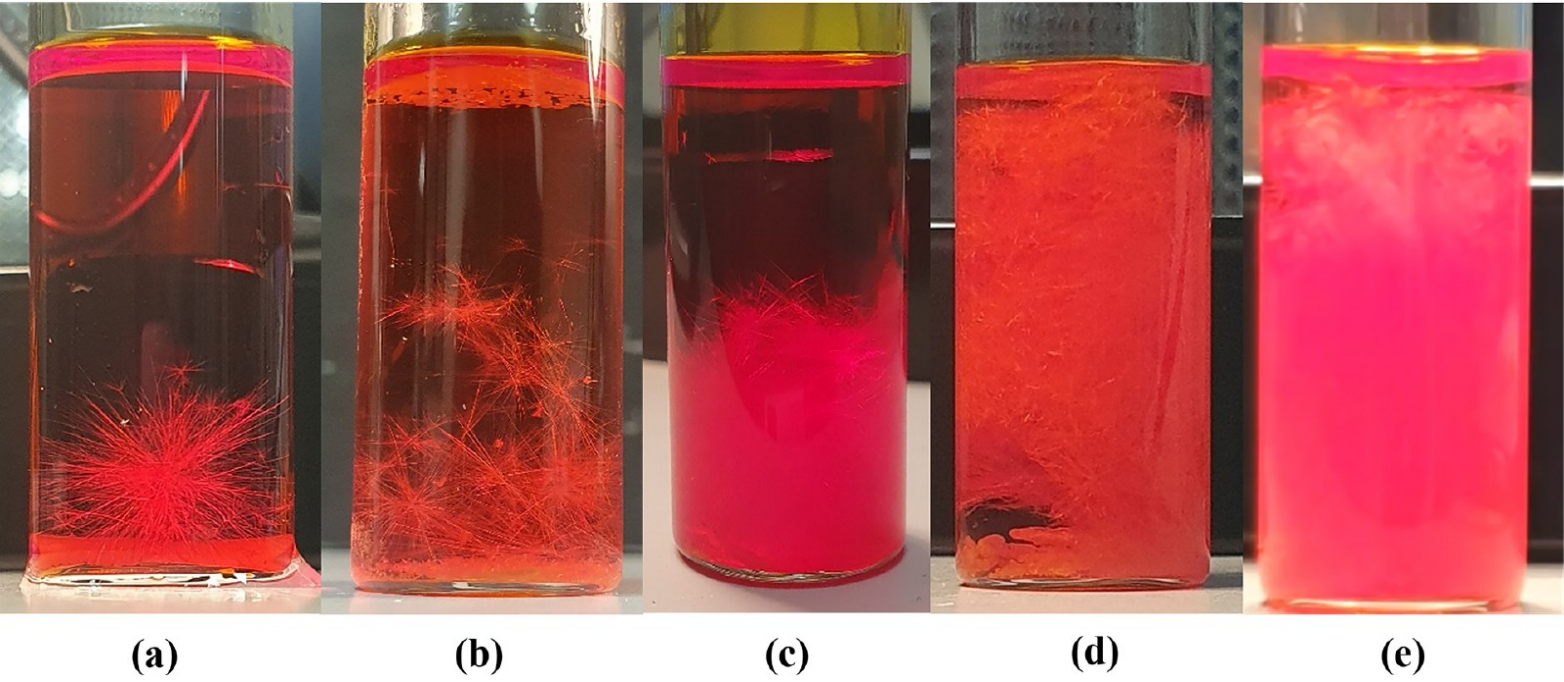
(a–d) ROY metastable polymorphs appearing after Y prism sedimentation and pipetting in the *c*/*c*_sat_ = 4.0 STFs: (a, b) YN crystals; (c, d) ON crystals. The image in (d) was taken several days later and shows the gradual growth of the sedimented Y prisms and slow disappearance of the ON form due to Ostwald ripening. (e) Visible clouding in a *c*/*c*_sat_ = 5.5 STF due to the emergence of the ROY amorphous form.

At higher supersaturation levels of *c*/*c*_sat_ = 5.5, crystallization was often preceded by the emergence of an amorphous form,^26^ evident from a visible clouding of the STF (Figure 7e). Optical microscopy revealed that this amorphous form consisted of ∼μm-sized droplets, which were subsequently consumed by crystalline forms via either Ostwald ripening (Figure 8) or direct impingement and incorporation into a growing crystal (Figure 9). The alternative explanation of the clouding resulting from a partial phase separation of the STF can be ruled out. Phase separation would proceed via either a rapid spinodal decomposition into two phase separated layers, which was never observed, or a slower nucleation and growth mechanism, which is inconsistent with the observations as these droplets did not grow and coalesce but instead dissolved or were consumed into crystals.

**Figure 8:**
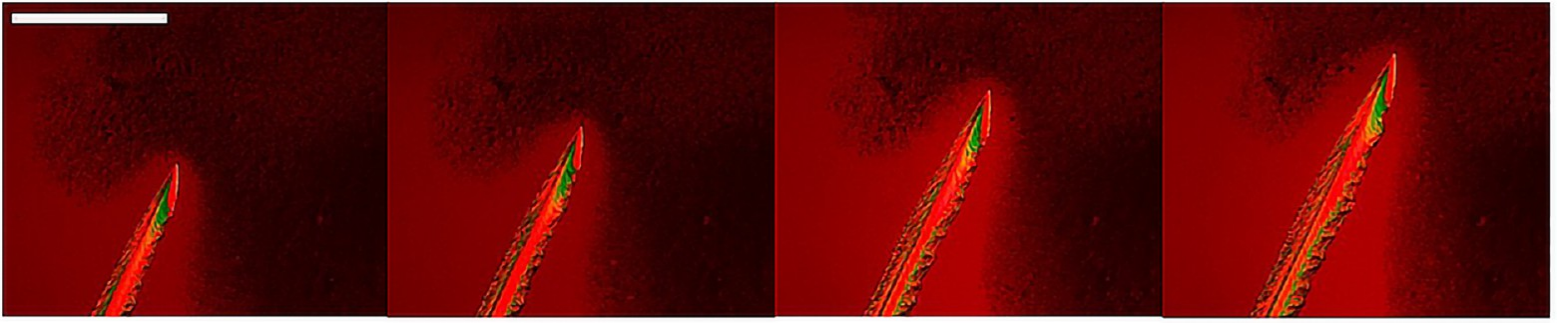
10 wt % toluene STF *c*/*c*_sat_ = 5.5, 10 s time-lapse images under crossed polars showing Ostwald ripening with consumption of the black amorphous droplet phase via dissolution around the growing ROY ON polymorph. Scale bar = 200 μm.

**Figure 9:**
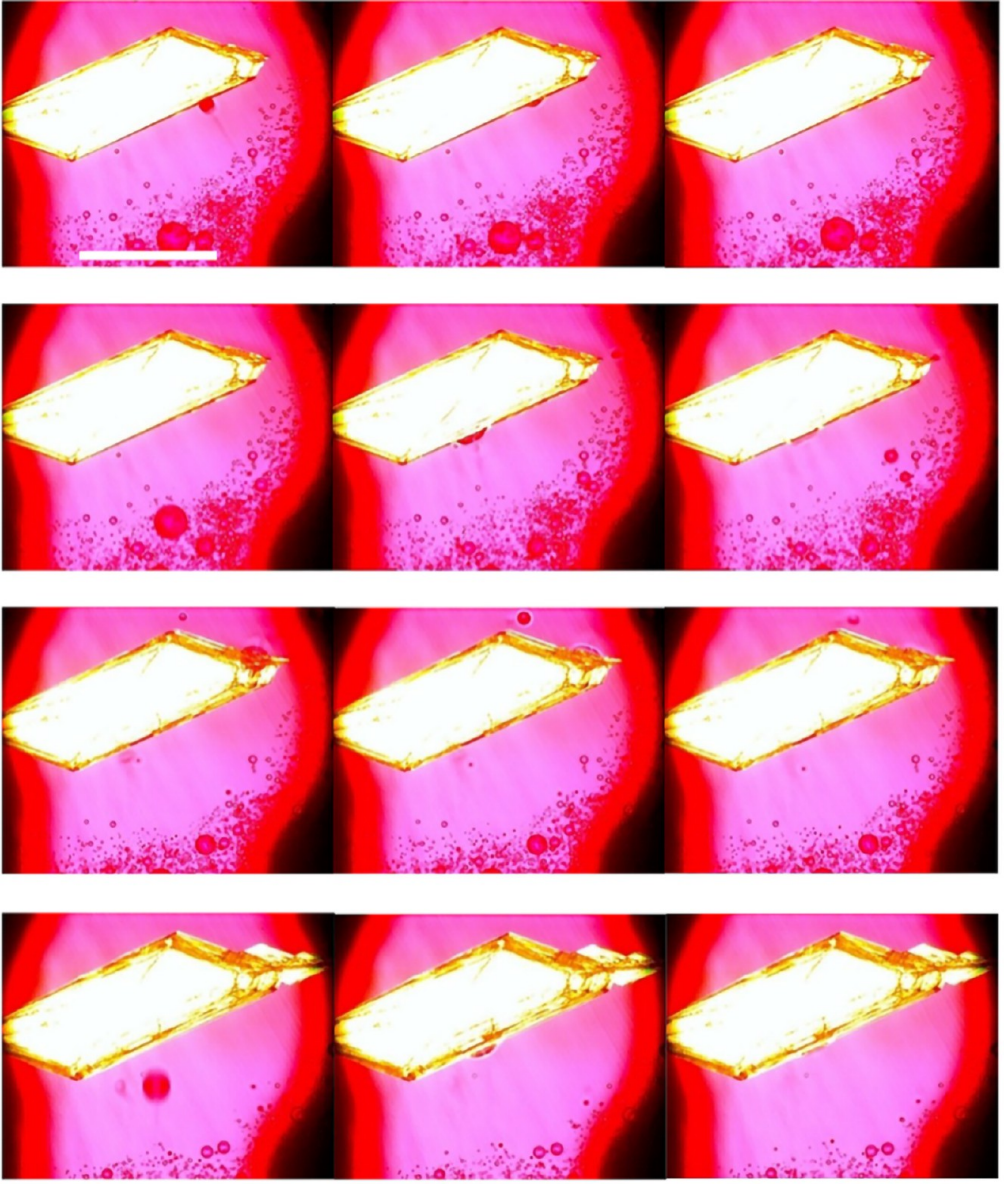
Crystal growth of a Y prism in the 20 wt % toluene STF with initial supersaturation of *c*/*c*_sat_ = 5.5. The images reveal Ostwald ripening via dissolution of the amorphous ROY droplets and amorphous droplet impingement and incorporation. Each row shows a different crystallization time, with consecutive images in the row taken 1 s apart. Images were taken under crossed polars with a red tint plate. Scale bar = 1 mm.

Significant quantities of the amorphous form produced a more viscous solution, incompatible with the previous pipetting STF disturbance, so for the *c*/*c*_sat_ = 5.5 experiments, only the rigorous shaking strategy was undertaken. Shaking was applied either 10 min after the appearance of clouding, or after 30 min if no clouding occurred. The results demonstrated that for these higher supersaturations, shaking significantly increased the fraction of metastable forms compared to the undisturbed controls (mean difference = +0.23, Tukey *post* *hoc* *p* = 0.0077) (Figure 10). This was most likely because the shaking was now conducted prior to significant Y prism nucleation so secondary nucleation of this form was suppressed. Instead, shaking provided energy to the amorphous droplets which aided nucleation of often several different polymorphs.

**Figure 10:**
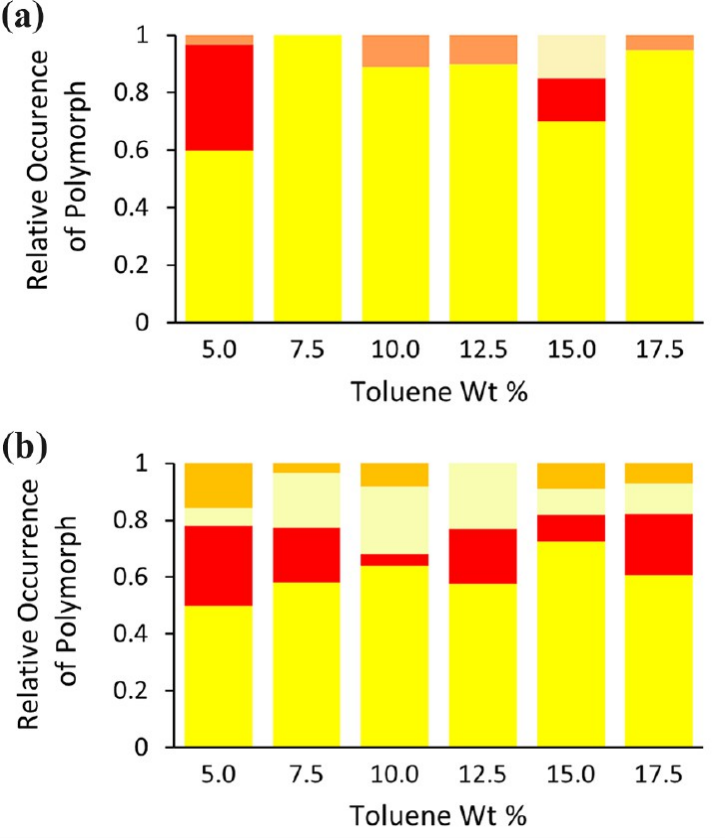
Relative occurrence of ROY polymorphs for 18 repeated crystallizations of ROY *c*/*c*_sat_ = 5.5 STFs for samples (a) left undisturbed and (b) shaken for 5 s after cloudiness was first observed or after 30 min if no cloudiness occurred in that time frame. Y prisms (yellow), YN (pale yellow), ON (orange), and R (red).

The ability to produce amorphous forms, metastable polymorphs and the stable polymorph of ROY from a single STF composition, despite the stable Y polymorph being the kinetic and thermodynamic product suggests STFs can provide a useful, low cost, easily accessible polymorph screening capability. This would complement recent high throughput screening strategies such as EnaCt (Encapsulated Nanodroplet Crystallization), which can provide an efficient and effective exploration of polymorphism but nevertheless rely on sophisticated equipment and crystallization from multiple (∼32) solvents.^19^

### 3.5 DLS *In Situ* Crystallization Studies

The course of ROY crystallization in the 5 wt % toluene STF was followed by DLS for a supersaturation level *c*/*c*_sat_ of 3.0 (Figure 11 and Figure S2). In Figure 11, the decay of the autocorrelation function, *g*_^(2)^_(*τ*)-1, with respect to delay time, *τ*, is initially representative of the diffusion of ∼1.5 nm-sized toluene-rich STF pockets. However, after the first 5 min run, the autocorrelation curve decays far more slowly, with a reduced gradient magnitude indicative of the presence of much larger particles of size ∼42 nm based on the standard cumulant analysis fitting. This is due to ROY crystallization producing suspended crystals in this size range. In subsequent runs, the STF 1.5 nm peak is largely invariant, whereas the suspended crystal peak varies as the crystals repeatedly nucleate, grow and sediment. After run 20, the quartz cuvette was removed from the DLS sample chamber and photographed to show the presence of Y prism sedimented crystals. Then, the STF was perturbed by the pipetting procedure and replaced in the DLS sample chamber. The last run and accompanying photograph in Figure 11 then show that significant secondary nucleation of ON occurred.

**Figure 11:**
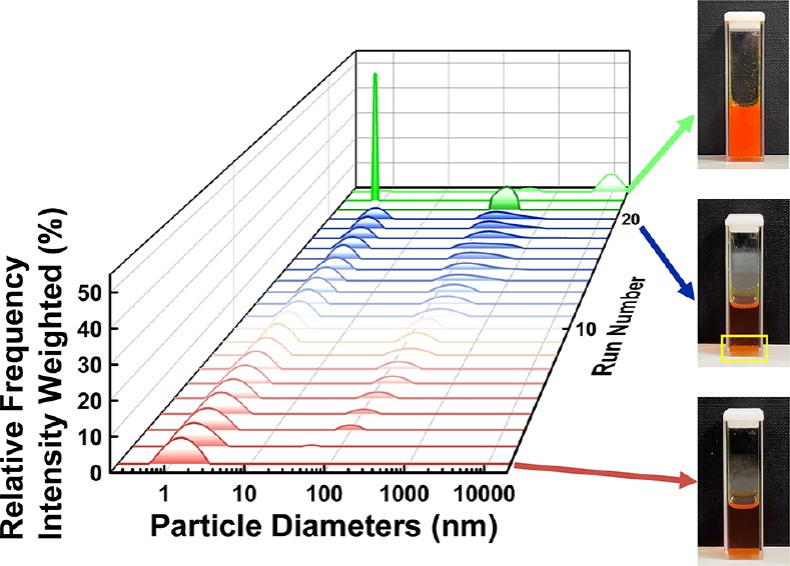
*In situ* ROY crystallization from 5 wt % toluene STF for *c*/*c*_sat_ = 3.0. Crystal nuclei of size >40 nm appear repeatedly from run 2. After run 20, the cuvette was removed from the DLS sample chamber to reveal Y prism crystals (middle photograph). Then, the STF was perturbed using the pipetting strategy and replaced in the DLS sample chamber. This led to secondary nucleation of ROY ON throughout the STF (top photograph), reducing the light transmission to low levels with a poor fit to the *g* ^(2)^(*τ*)-1 curve due to the rapidly changing environment.

Note that the intensity of the scattered radiation scales as particle size to the power of 6, since Rayleigh scattering intensity depends upon the square of the particle polarizability and so the particle volume squared. Hence, the relative number of crystal nuclei compared to STF nm-sized pockets can be readily estimated. For instance, in run 2, the fraction of ∼42 nm nuclei to ∼1.5 nm STF sites is ∼0.02 × (1.5/42)^6^ ∼ 4 × 10^–11^ given the relative peak areas are 1:49. Assuming that the majority of toluene is confined to droplets comprised of ∼50 wt % toluene and ∼50 wt % IPA since IPA will be distributed fairly evenly between aqueous and toluene rich domains, with a slight excess at the interface between the two, then the number of toluene-containing droplets is ∼10^20^ per cm^3^ of STF. This leads to an average nucleation rate during the 5 min of run 2 of ∼1 × 10^7^ cm^–3^ s^–1^ with ∼1 × 10^–13^ of the pockets acting as nucleation sites per second.

In the second *c*/*c*_sat_ = 3.0 crystallization, suspended crystals of size ≥40 nm do not emerge until run 8 (Figure S2). Notably, though for run 7, the STF peak becomes unsymmetrical, due to broadening on the larger length scale side, suggesting either the growth of some droplets, or the first emergence of small ROY nuclei. In run 9, only the STF peak remains, as the suspended crystals in the previous run have grown and sedimented. Further ROY nucleation is then evident in run 15, with sporadic nucleation, growth and sedimentation of crystals continuing for extended time periods after this. These DLS studies therefore confirm that nucleation and crystal growth times are extended in STFs compared to bulk solution; in bulk solution, the supersaturation is depleted to levels impeding further nucleation rapidly due to unrestricted diffusion and so faster crystal growth. Consequently, the longer nucleation time frame and slower crystal growth make STF systems ideal for early stage crystallization studies.

## 4 Conclusions

In conclusion, we propose a crystallization model in STFs where nucleation of kinetically unfavored nuclei can occur under prolonged, locally high supersaturation conditions, enabled by the nanoconfinement in the STF. The greater the nanoconfinement, the greater the likely supersaturation variation and the slower the crystal growth. Furthermore, greater nanoconfinement increases the probability of metastable polymorphs surviving alongside more stable ones, due to reduced Ostwald ripening rates from restricted diffusion. To further promote access to ROY metastable polymorphs, a strategy of disturbing the STF, particularly by pipetting after sedimentation of the faster-growing Y prisms, proves advantageous. This pipetting perturbation increases autocatalytic secondary nucleation of any suspended nuclei, selectively promoting the formation of more metastable polymorphs. Hence, STFs can facilitate detection of even slow-growing metastable forms, particularly when combined with appropriately timed local perturbation of the STF by pipetting, following sedimentation of the faster growing form.

## Supplementary Material















## Notes

ROY solubility data, STF droplet sizes, relative crystal growth rates, DLS *in situ* ROY crystallization, and viscosity data (PDF)
Video S1: Comparative ROY crystal growth rate for high supersaturation 5 wt % toluene STF and Binary system (AVI)
Video S2: Comparative ROY crystal growth rate for high supersaturation 5 and 20 wt % toluene STFs (AVI)
Video S3: Comparative ROY crystal growth rate for high supersaturation 20 wt % toluene STF and Binary system (AVI)
Video S4: Comparative ROY crystal growth rate for lower supersaturation 5 wt % toluene STF and Binary system (AVI)
Video S5: Comparative ROY crystal growth rate for lower supersaturation 5 and 20 wt % toluene STFs (AVI)
Video S6: Comparative ROY crystal growth rate for lower supersaturation 20 wt % toluene STF and Binary system (AVI)

## †

Present Address: Leiden Institute of Chemistry, Leiden University, Einsteinweg 55, 2333 CC Leiden, The Netherlands (N.L.F.)
