# Supplementary material for: Structured Ternary Fluids Promote Crystallization and Detection of 5‑Methyl-2-[(2-nitrophenyl)amino]-3-thiophenecarbonitrile (ROY) Metastable Polymorphs
Source: Cryst Growth Des. 2025 Nov 4;25(22):9692–701. doi: 10.1021/acs.cgd.5c00838 (PMC12636027; doi:10.1021/acs.cgd.5c00838)
Supplement: Supplementary file 1 [file cg5c00838_si_001.pdf]

Supporting Information for

# Structured Ternary Fluids Promote Crystallization and Detection of ROY Metastable Polymorphs

*Yang Shu, Jennifer J. Maunder, Nicci L. Fröhlich, Madeleine Rolle-Binne and Sharon J. Cooper*

Department of Chemistry, Durham University, Durham, DH1 3LE, UK

**Table S1**. Solubility of ROY in STFs based on amount dissolving in 2 months.

| Wt % toluene in STF | Solubility in 1 g of STF / mg | |
|---|---|---|
| | 7˚C | 25˚C |
| 5 | 0.88 | 2.2 |
| 7.5 | 0.55 | 3.5 |
| 10 | 1.12 | 4 |
| 12.5 | 1.99 | 6.2 |
| 15 | 3.11 | 8.1 |
| 17.5 | 3.51 | 9.6 |
| 20 | 4.12 | 10 |
| 27.5 | 7.41 | 16.9 |
| 32.5 | 8.19 | 18.5 |
| 37.5 | 9.39 | 23 |
| 40 | 9.56 | 26.6 |
| 47.5† | 10.27 | 30.5 |

† Toluene:IPA binary solution

**Table S2**. Estimated STF droplet sizes from standard cumulant analysis fitting of the $g^2(\tau)$-1 curve using the standard cumulant analysis method.

| No. † | Water / % | Toluene / % | IPA / % | DLS Droplet Diameters / nm ‡ |
|---|---|---|---|---|
| 1 | 47.5 | 0 | 52.5 | $0.74 \pm 0.08$ |
| 2 | 42.5 | 5 | 52.5 | $1.05 \pm 0.02$ |
| 3 | 37.5 | 10 | 52.5 | $1.59 \pm 0.01$ |
| 4 | 32.5 | 15 | 52.5 | $2.30 \pm 0.02$ |
| 5 | 27.5 | 20 | 52.5 | $2.56 \pm 0.02$ |
| 6 | 22.5 | 25 | 52.5 | $2.33 \pm 0.03$ |
| 7 | 17.5 | 30 | 52.5 | $1.64 \pm 0.03$ |
| 8 | 12.5 | 35 | 52.5 | $1.03 \pm 0.02$ |
| 9 | 7.5 | 40 | 52.5 | $0.72 \pm 0.04$ |
| 10 | 2.5 | 45 | 52.5 | $0.56 \pm 0.02$ |
| 11 | 0 | 47.5 | 52.5 | $0.50 \pm 0.03$ |

†The number corresponds to the same composition numbering in Figure 1. Measurements were repeated 30 times per sample. Standard deviations are reported as the measure of uncertainty, with the low values indicating consistent fitting.

‡The DLS-derived droplet sizes correspond to the hydrodynamic diameters of spheres diffusing at the same rate as the STF nanopockets. However, the actual STF nanopocket shape will deviate from spherical.

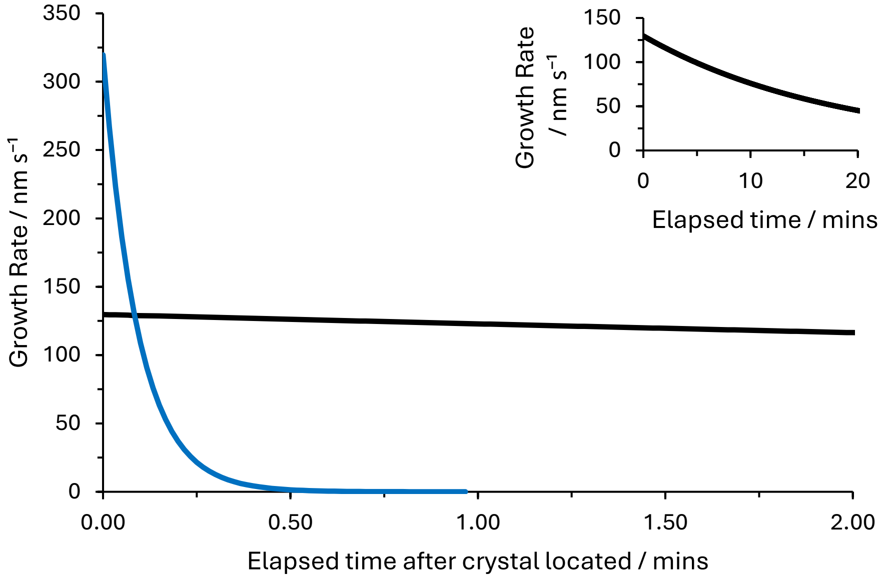


**Figure S1**. Relative growth rates of a ROY Y prism crystal in the 5 wt % toluene STF (black) compared to the binary 47.5 wt % toluene:52.5 wt % IPA system (blue) at $c/c_{sat}$ = 5.5. The inset shows crystal growth occurs for significantly greater times periods in the STF compared to the binary system. The growth rate data was obtained from the images used in movie S1.

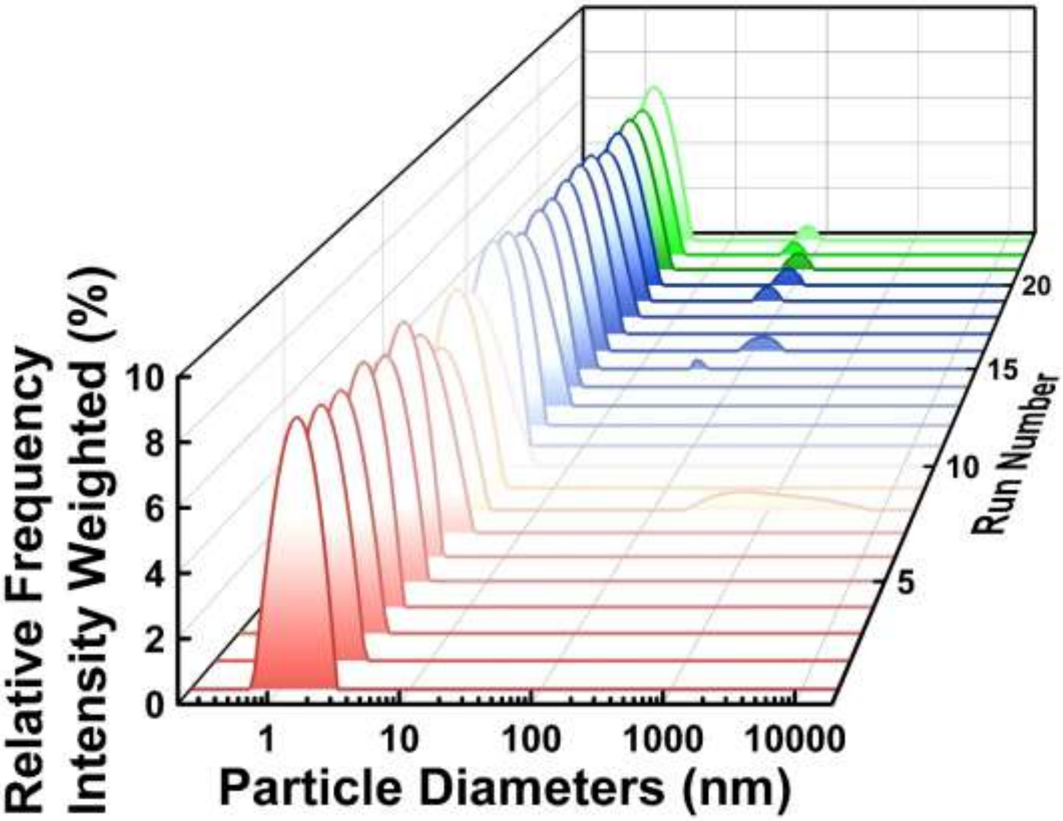


**Figure S2**. *In situ* ROY Y prism crystallization from 5 wt % toluene STF for $c/c_{sat}$ = 3.0. Crystal nuclei of size > 40 nm appear repeatedly from run 8, grow and then sediment to leave just the STF peak at ~1.5 nm again. Note in runs 7 and 10, the STF peak has extended on the longer length side, which may be due to the presence of small ROY nuclei in this size range.

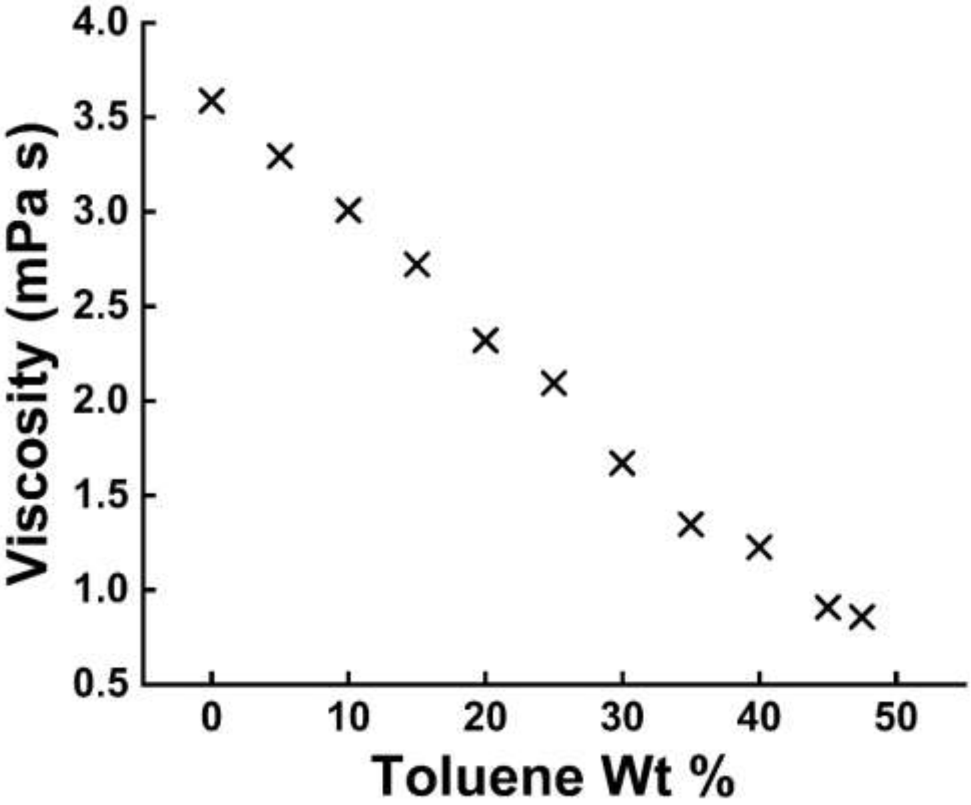


**Figure S3**. Viscosities of the STFs and toluene:IPA binary solution.
